# Physical-Chemical and Structural Stability of Poly(3HB-co-3HV)/(ligno-)cellulosic Fibre-Based Biocomposites over Successive Dishwashing Cycles

**DOI:** 10.3390/membranes12020127

**Published:** 2022-01-21

**Authors:** Estelle Doineau, Fleur Rol, Nathalie Gontard, Hélène Angellier-Coussy

**Affiliations:** JRU IATE 1208, INRAE, Montpellier SupAgro, University of Montpellier, CEDEX 02, 34 060 Montpellier, France; estelle.doineau@umontpellier.fr (E.D.); fleur.rol15@gmail.com (F.R.); nathalie.gontard@inra.fr (N.G.)

**Keywords:** PHBV, stability, ageing, packaging, dishwasher, biocomposites

## Abstract

In order to lengthen the life cycle of packaging materials, it is essential to study their potential for reuse. This has been never carried out for emerging bio-based and biodegradable materials such as PHBV/(ligno-)cellulosic fibre-based biocomposite materials. This work therefore highlights the impact of successive dishwashing cycles on the physical-chemical and structural stability of such materials. Several parameters were considered to assess this stability, such as the visual aspect and colour, the microstructure, the thermal and tensile properties, and the overall migration in food liquid simulants. The effect of fibre composition, morphology, and content was investigated by selecting three types of commercial (ligno-)cellulosic fibres and two filler contents (20 and 40 wt%). A great potential for reuse of PHBV films was highlighted by their high stability after up to at least 50 dishwashing cycles. However, the addition of (ligno-)cellulosic fillers negatively impacts the stability of PHBV-based materials, especially due to the hygroscopic behaviour of (ligno-)cellulosic fillers and the heterogenous microstructure of biocomposites, with at best up to 10 possible dishwashing cycles for ultra-pure cellulose. In conclusion, reuse including dishwashing steps can be considered for neat PHBV materials, while this should be prohibited for PHBV/(ligno-)cellulosic fibre-based biocomposite materials.

## 1. Introduction

Plastic wastes are one of the causes of current environmental concerns, targeting in particular packaging applications, including single-use packaging such as disposable dishes [1,2,3]. Indeed, despite efforts to collect and recycle plastic wastes, such non-biodegradable wastes imply a huge amount of plastic microparticles disseminated in all the compartments of our environment [4,5], being harmful to all ecosystems [6]. The substitution of non-biodegradable oil-based plastic packaging by bio-circular materials stemming from biomass, without competition with food usages, and able to go back to the soil through biodegradation or composting is currently the only valuable mean to address the overwhelming negative impacts of our plastic wastes while entering a virtuous closed-loop regenerative system [7]. In this sense, in order to minimize the use of resources, it is essential to lengthen the life cycle of packaging materials via several recycling closed loops [8] and/or reuse steps [9], before an ultimate composting and/or biodegradation step. Recent European Union decrees go in this direction, e.g., with the article L. 541-10-17 of the Environmental code requiring a 20% reduction target for single-use plastic packaging set for producers, of which at least 50% must be obtained by reuse by 31 December 2025 [10]. Moreover, it is important to keep in mind that biodegradable and/or composting notions are not incompatible with recycling and reuse processes. Today, the vast majority of consumers are still confused about these terms with the feeling that compostable packaging can only be used once.

Among biodegradable polymers, polyhydroxyalkanoates (PHAs) are thermoplastic bacterial polyesters which have recently received a great interest from the scientific community [11,12,13], especially the semi-crystalline poly(3-hydroxybutyrate-co-3-hydroxyvalerate) (PHBV). Indeed, the incorporation of the hydroxyvalerate (HV) monomers in the fermentation process, in comparison to poly(3-hydroxybutyrate) (PHB), gives higher flexibility and better processability to the polymer [14]. The stability of such biopolymers has been mostly investigated in terms of ageing and biodegradability in indoor conditions under different environmental conditions (e.g., temperature, medium such as soil, compost, water or marine water) [15,16,17,18,19]. In a previous work, new insights into the physical-chemical and structural stability of PHBV films under food contact conditions have been gained [20]. It was shown that the functional properties of PHBV films (mechanical properties and water vapour permeability) remained very stable after 10 days of contact at 40 °C with all tested food simulating liquids, except with ethanol 95% (*vol*/*vol*). In this case, a high sorption value together with a significant plasticizing (swelling) effect and an increase in the water vapour permeability were noticed, which was mainly explained by a decrease in both the molecular weight and the crystallinity degree of PHBV films.

Very few studies have focused on the potential for reuse and/or recycling of PHB copolymer or PHBV-based packaging items, with articles mainly focusing on the mechanical recycling [21,22,23,24]. Since the reuse implies successive steps of usage and washing, the stability of packaging items should be guaranteed throughout the process. Both at the consumer and industrial levels, the washing of reusable materials such as glass is mainly carried out in dishwashers, involving the use of hot water and chemicals. A standard has been set up (NF EN 12875-1) to define the dishwashing testing conditions used to consider whether a product is reusable or not. So far, although PHBV-based materials are authorized for food contact applications by the European Plastics Regulation (EU) N° 10/2011 [25], there is no article dealing with the assessment of the stability of PHBV-based materials over successive dishwashing cycles.

In parallel, the use of lignocellulosic fibres in biocomposites in various sectors has many advantages, such as their biodegradability, wide availability, renewability, low cost, and low density [26,27,28]. The incorporation of lignocellulosic particles in composites can play the role of reinforcements by increasing mechanical properties, particularly the Young’s modulus and strength, and/or play the role of fillers to reduce the overall cost and environmental impact, while providing new functionalities to the material. In the present work, (ligno-)cellulosic fillers were added to PHBV to address the second issue mentioned above [29,30,31]. Studies on the biodegradation of such PHBV-based biocomposites showed that lignocellulosic fibres did not compromise the biodegradation of PHBV polymer [15,32]. However, the polyphenol content of lignocellulosic fibres had an impact on the biodegradation kinetics with an acceleration or slowing down. It was shown that the incorporation of micrometric-sized lignocellulosic fibres in a PHBV matrix impacted its stability in food contact conditions, especially in aqueous media, mainly due to the hygroscopic character of lignocellulosic fibres and to the leaching of water-extractable components [29]. It was demonstrated that PHBV-based composite materials could be used in contact with food products displaying a water activity equal or lower than 0.90.

Whereas the demand of the food packaging sector for PHBV-based biocomposite materials is increasing, their potential for reuse as food containers by using a dishwasher was not investigated in the literature, rather focused on biodegradation in air, in water, or in compost environments [15,19,33,34] or on conventional polymers such as PET [35]. In this context, the goal of this work was to assess the impact of successive dishwashing cycles on the structural and physical-chemical stability of PHBV/(ligno-)cellulosic fibre-based biocomposite materials. The effect of fibre composition, morphology, and content was investigated by selecting three types of commercial (ligno-)cellulosic fibres and two filler contents (20 and 40 wt%). For that purpose, PHBV-based films were produced by melt extrusion followed by a thermopressing step. In accordance with the standard NF EN 12875-1, up to 125 dishwashing cycles were carried out. Films having undergone a certain number of cycles in the dishwasher have been characterised under standardised testing conditions. The evolution of the colour, the tensile properties, and the overall migration in two food liquid simulants, i.e., ethanol 50% (*vol*/*vol*) and ethanol 95% (*vol*/*vol*), was discussed in relation to the structural stability of materials, which was evaluated by changes in sample weight and thickness, thermal transition temperature, and crystallinity assessed by differential scanning calorimetry (DSC), as well as microstructures observed by scanning electron microscopy (SEM).

## 2. Materials and Methods

### 2.1. Materials

Poly(3-hydroxybutyrate-co-3-hydroxyvalerate) (PHBV) with 3% HV (molar content) was purchased from Natureplast under the grade PHI003, in the form of a fine and electrostatic powder (d_50_ = 19.9 ± 2.3 µm). Boron nitride was purchased from Sigma Aldrich (reference 255475) and used as nucleating agent. Three different grades of (ligno-)cellulosic fibres were supplied by J. Rettenmaier & Söhne (JRS), i.e., Arbocel B00, Arbocel BE 600 30 PU and Filtracel EFC 950. Their intrinsic characteristics are fully detailed in the Section 3. Results and Discussion, e.g., Section 3.2.1. Description of (ligno-)cellulosic fillers.

### 2.2. Production of PHBV-Based Films

Compounding. The PHBV powder was first mixed with 0.5 wt% of boron nitride (BN) in a plastic bag for 5 min at 23 rpm using a turbulator (Turbula T10B Laboservices, France). The mixture was then dried at 60 °C during 24 h before compounding in a twin-screw extruder (Thermoscientific Prism Eurolab 16XL) with a temperature profile from the feeding to the filament die of 140–150–160–160–170–170–180–180–180 °C, a flow rate of 1 kg.h^−1^ and a screw speed fixed at 200 rpm. Due to the electrostatic character of the powder, it was manually introduced as steadily as possible. The resulting compound was pelletized and dried at 60 °C during 24 h. Composite compounds were then produced using the same processing conditions. A single screw volumetric feeder, 16 mm (Brabender DSR28, Germany) and a twin screw gravimetric feeder (Brabender DDW-MD1-MT2, Germany) were used, respectively, for the matrix pellets and the (ligno-)cellulosic fibres. Fibres were dried at least 24 h at 60 °C before compounding and were added at 20 and 40 wt%. Obtained biocomposites were pelletized, dried at 60 °C during 24 h and stored in hermetic bags. In order to homogenise the nomenclature for composites, they will be referred to as such in the following: *PHBV/X wt% filler code*, with *X* the weight filler content and the filler code given in Table 2. For example, *PHBV/20 wt% BE 600* corresponds to PHBV filled with 20 wt% of Arbocel BE 600 30 PU fillers.

Film production. Biocomposite compounds were dried at 60 °C during 24 h and then shaped in films by thermopressing using a heated hydraulic press (20T, Pinette Emidecau Industries). 6 g of pellets were heated between two Teflon-coated plates in a 300 µm sieve to obtain films of 12 × 12 cm dimensions following 5 processing steps: contact with plates at 180 °C for 5 min, then pressing at 180 °C for 30 s under 50 bar, followed by pressing at 180 °C for 30 s under 100 bar, then pressing at 180 °C for 60 s under 150 bar and finally cooling at ambient temperature using a 1 kg weight. Produced composite films were then stabilized in a conditioning room at 25 °C and 50% HR for a minimum of 14 days before further characterisation.

### 2.3. Dishwashing Procedure

**Figure 1:**
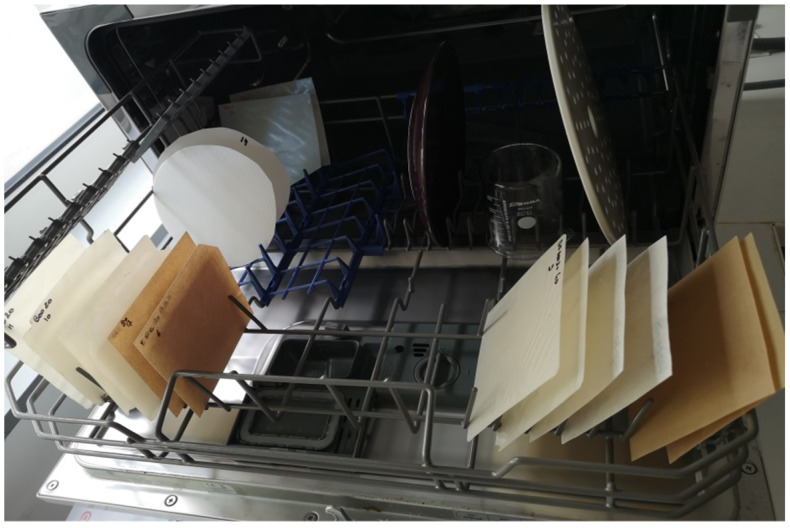
Dishwasher containing PHBV-based films.

In accordance with the standard NF EN 12875-1, the required number of dishwashing cycles needed to consider a product is reusable is 125. Up to 125 cycles were thus applied to neat PHBV films. In the case of biocomposite films, only 10 successive cycles were applied, due to the instability of materials. First, film thickness was measured with a micrometer at three different locations and then films were placed in a commercial dishwasher (ELVC-491b, Essentielb) (Figure 1). In accordance with the standard NF EN 12875-1, the dishwasher was completely filled before starting the cycle.

Commercial dishwashing products were used, i.e., cleaning agent (SUN maxi tablets 3 in 1), rinsing agent (SUN triple action rinsing) and regenerating salt (SUN classic regenerating salt) to prevent limescale build-up. The overall dishwashing cycle lasted 90 min, including a washing step at 65 °C, a rinsing step at 70 °C, and a drying step around 80–90 °C. After each cycle, PHBV-based films were manually wiped. Samples were recovered at given intervals for further analysis, i.e., after 1, 3, 5, 10, 45, 50, 70, 100, and 125 cycles for neat PHBV films and after 1, 3, 5, and 10 cycles for biocomposites. Samples were dried at 60 °C during 24 h and then stabilized in a conditioning room at 25 °C and 50% HR for a minimum of 14 days before further characterisation.

### 2.4. Laser Granulometry

A laser granulometer (Malvern Mastersizer 2000 Instrument Ltd, United Kingdom) was used in the wet mode to determine the fibre size distribution. Fibres were progressively added to ethanol 95% (*vol*/*vol*) until reaching the required concentration by the granulometer. From the particle size distribution in volume, four values were identified to characterise the sample: d_10_, which represents the apparent diameter for which 10% of particles have a diameter below, d_50_, which represents the apparent median diameter, d_90_, which is the apparent diameter from where the cumulative distribution of particles is 90%, and the span defined as (d_90_ − d_10_)/d_50_ and representing the width of the size distribution. Calculations were done based on the assumption that the particles are spherical.

### 2.5. Colorimetry

The colour attributes of PHBV-based films were measured with a Konica Minolta CR-410 colorimeter (Nieuwegein, The Netherlands), using the CIELAB colour system (L*, a*, b*). L* represents the luminance, ranging from 0 (black) to 100 (white), while the two chromatic components a* (from green to red) and b* (from blue to yellow), range from −120 to +120. For each formulation and after each dishwashing cycle, the total colour difference (ΔE), defined by the Commission Internationale de l’Eclairage (CIE) [36], was calculated by using as reference the film before any dishwashing step (cycle 0) (Equation (1)):

(1) $$∆E=\sqrt{{\left(L_{i}^{*}-L_{0}^{*}\right)}^{2}+{\left(a_{i}^{*}-a_{0}^{*}\right)}^{2}+{\left(b_{i}^{*}-b_{0}^{*}\right)}^{2}}$$

where L*, a* and b* are the colour components of each sample, and *i* the number of dishwashing cycles. Colour differences were evaluated as follows: ΔE ≤ 1: not visible; 1 ≤ ΔE ≤ 3.5: poorly visible; 3.5 ≤ ΔE ≤ 5: clearly noticeable; ΔE ≥ 5: different colours are notable [37].

### 2.6. Differential Scanning Calorimetry (DSC)

Differential Scanning Calorimetry (DSC) tests were carried out using a Q200 equipment (TA Instruments, New Castle, DE, USA) with nitrogen as purge gas (50 mL.min^−1^). Samples of about 10 mg were cut from the films and placed in hermetic aluminium pans (TA Instruments, New Castle, DE, USA). The thermal cycle was a first heating from 20 °C to 200 °C, then an isotherm at 200 °C during 5 min, then a cooling down to −30 °C and a second heating up to 200 °C, with heating/cooling rates of 10 °C.min^−1^. The degree of crystallinity *χ_c_* (%) was calculated according to the following Equation (2):

(2) $$\chi_{c}=100\times \frac{∆H_{m}\;}{\left(x_{matrix}\times \;∆H_{m}^{0}\right)}$$

With Δ*H_m_* (J.g^−1^) the melting enthalpy of the first heating step of the sample (J.g^−1^), *x_matrix_* the weight fraction of PHBV within the sample and Δ*H_m_*^0^ is the melting enthalpy of 100% crystalline PHB (146 J.g^−1^) [38]. Experiments were done in duplicate.

### 2.7. Tensile Properties

Tensile tests were carried out at room temperature using a texture analyser machine (Zwick BZ2.5/TN1S, France) equipped with a force sensor (KAFS, A.S.T, Germany) at a speed of 1 mm.min^−1^, on samples cut in dumbbell shape with a width of 4 mm, a length L_0_ = 22 mm of the effective cross-section and a distance between jaws of 45 mm. At least five tests were done for each sample.

### 2.8. Overall Migration

Overall migration (OM) tests were evaluated according to the European Standard EN 1186 series. Tests were carried out at 40 °C during 10 days in two selected food liquid simulants, i.e., ethanol 50% (*vol*/*vol*), to simulate juices, food preserved in alcohol, eggs and dairy products [25], and ethanol 95% (*vol*/*vol*) considered as the worst case of PHBV [20]. In order to provide a ratio of 6 dm^2^.L^−1^ between the film and the food simulant liquid, six disks of 2.5 cm diameter were introduced in 100 mL in a migration cell. Before immersion, disks were dried at 60 °C during 48 h. After immersion, disks were dried at 60 °C during at least 48 h until reaching a steady mass. The overall migration value was calculated as follows (Equation (3)):

(3) $$\mathrm{Overall}\;\mathrm{migration}=\frac{m_{film,\;\;\;before\;contact}-m_{film,\;\;\;after\;contact}\;}{surface\;in\;contact}$$

with the overall migration in mg.dm^−2^, *m* in mg and the *surface in contact* in dm^2^.

## 3. Results and Discussion

### 3.1. Stability of PHBV

#### 3.1.1. Visual Aspect and Colour

**Figure 2:**
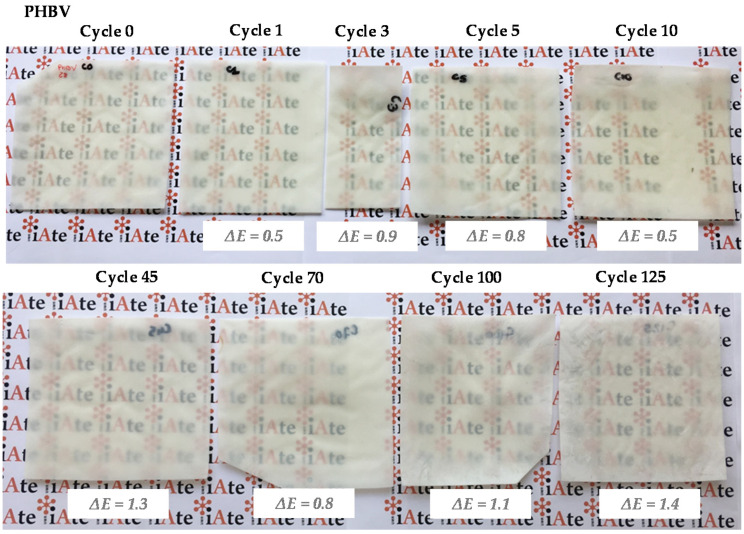
Pictures of PHBV films having undergone up to 125 dishwashing cycles with total colour difference values ΔE calculated by using as reference the film before any dishwashing step (cycle 0).

Pictures of PHBV films were taken going from 0 to 125 dishwashing cycles in order to see potential visual differences in terms of optical properties (colour, transparency and bleaching), surface roughness, and mechanical integrity (Figure 2). No clear visual change in optical properties was noticed up to 125 cycles of dishwashing. The calculation of the total colour difference (ΔE) confirmed that colorimetric differences were not visible or only poorly visible (in case of ΔE values higher than 1), with values ranging from 0.3 to 1.4. Transparency was also preserved, which likely reflected little or no change in crystallinity. However, it has to be mentioned that films were more damaged with either folds or missing corners from around 50 dishwashing cycles. This could be due to an increased brittleness, as further investigated by tensile tests.

#### 3.1.2. Tensile Properties

**Figure 3:**
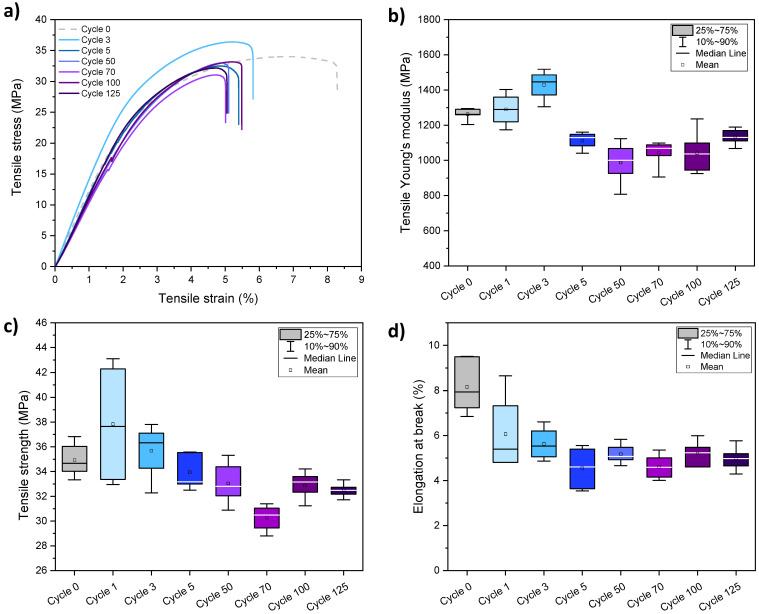
Typical uniaxial tensile curves (**a**), Young’s modulus (**b**), tensile strength (**c**) and elongation at break (**d**) of PHBV films having undergone up to 125 dishwashing cycles.

Tensile properties were measured on PHBV films having undergone up to 125 dishwashing cycles. All samples displayed a mechanical behaviour typical of a rigid thermoplastic polymer (Figure 3a). The tensile mechanical characteristics, i.e., the elongation at break, the tensile strength and the Young’s modulus, were not impacted similarly upon successive dishwashing cycles. On the first hand, the elongation at break of PHBV films (Figure 3d) dropped from about 8% to 5% during the first 5 cycles, and then remained constant up to 125 cycles, confirming the increased brittleness previously visually assessed. On the other hand, the Young’s modulus (Figure 3b) and tensile strength (Figure 3c) first increased by 13.5% and 8.7% respectively during the first three cycles of dishwashing, and then decreased during the next two cycles, to reach equilibrium values of 1.1 GPa and 32 MPa, respectively. Globally, a downward trend for all mechanical properties was observed after five dishwashing cycles, and after 125 cycles all mechanical properties have significantly decreased, i.e., Young’s modulus by 11.5%, the tensile strength by 5.8% and the elongation at break by 38.5%. The tensile strength was less impacted than other properties. 

Similar behaviour was observed by Deroiné et al. who studied the accelerated ageing of PHBV films in distilled water at different temperatures (25, 30, 40 and 50 °C) during one year [16]. Such behaviour is characteristic of an embrittlement of PHBV, which was ascribed to the hydrolysis of ester bonds, inducing a decrease of the molecular weight of PHBV. Authors mentioned that an increase of temperature implied an increase of water diffusivity within the polymer, and so an increase of the hydrolysis phenomenon. It has to be noticed that in our case, conditions were harsher with higher temperatures (the dishwashing cycle included a washing step at 65 °C, a rinsing step at 70 °C and a short drying step around 80–90 °C), the presence of a mechanical water circulation and also the use of dishwashing products within the dishwasher. Hydrolysis reactions could occur both at the surface and in the bulk of the PHBV films, given the observed weight uptake (from 0.4% to 1.1% over the first 10 cycles) that was attributed to water uptake, detailed later in this article (Figure 8a). 

The increase of PHBV’s stiffness observed over the first three dishwashing cycles (Figure 3b) highlights a densification of the material, which could be explained by possible condensation reactions occurring in amorphous parts of PHBV. After five cycles, a strong decrease of mechanical properties is observed and could be ascribed to hydrolysis reactions already mentioned in the work of Deroiné et al. [16]. It could be that the two phenomena, i.e., condensation and hydrolysis reactions, are in competition during the first three dishwashing cycles and then hydrolysis reactions become dominant in the PHBV material.

It was demonstrated that changes in mechanical behaviour could not be ascribed to changes in the overall crystallinity of materials, as shown by DSC results (Table 1). Globally, thermal properties (i.e., melting and crystallization temperatures) were not significantly impacted by successive dishwashing cycles.

**Figure 4:**
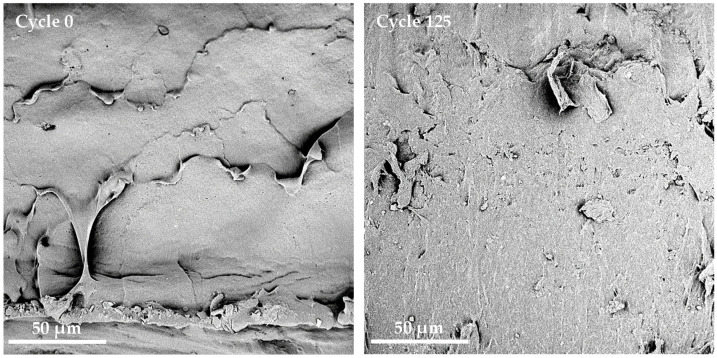
SEM images of cryofractured cross-sections of PHBV films having undergone 0 or 125 dishwashing cycles.

To complete the microstructure analysis, SEM images were taken on cryofractured cross-sections on PHBV films at cycle 0 or after 125 dishwashing cycles (Figure 4). Although the crystallinity was not impacted by successive dishwashing cycles (Table 1), some differences were observed on SEM pictures. After 125 cycles of dishwashing, the cross-section appeared smoother at the macroscopic scale, suggesting a more fragile fracture during the preparation of the sample. This could be correlated with the increase of the brittleness of PHBV films after 125 dishwashing cycles highlighted by tensile tests (Figure 3). At a more local scale, some irregularities were visible with the apparition of thin white trails, probably due to the penetration of water within the film.

#### 3.1.3. Overall Migration in Liquid Food Simulants

**Figure 5:**
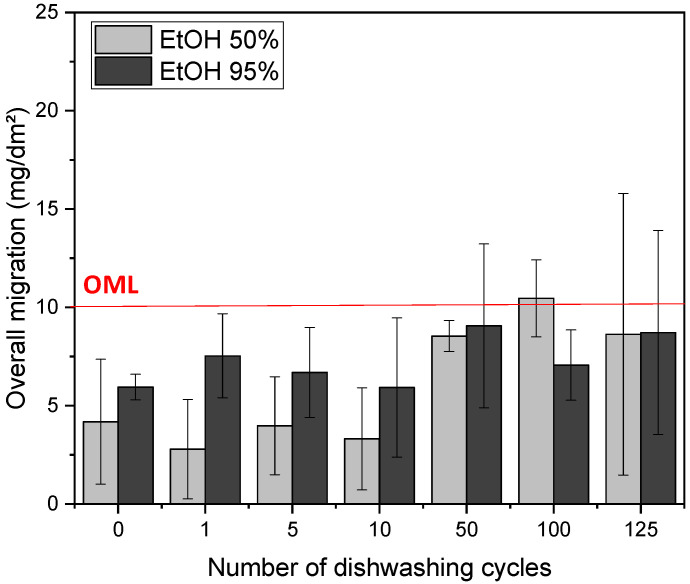
Overall migration values of PHBV films in ethanol 50% (*vol*/*vol*) and ethanol 95% (*vol*/*vol*) after a given number of successive dishwashing cycles. The red line corresponds to the overall migration limit (OML) set by the European Commission (2011) [25].

The overall migration (OM) under food contact represents the quantity of non-volatile substances liberated by the material in a food simulant under given conditions of temperature and duration. Overall migration tests have been carried out using two different liquid food simulants, i.e., ethanol 50% (*vol*/*vol*) and ethanol 95% (*vol*/*vol*), in order to evaluate their ability for food contact applications (Figure 5). The overall migration limit (OML) has been set at 10 mg.dm^−2^ by the European Commission [25].

In the case of ethanol 95% (*vol*/*vol*), OM was not significantly affected by successive dishwashing cycles, with values ranging from 5.9 ± 3.5 to 9.1 ± 4.2 mg.dm^−2^ and remaining lower than the OML, demonstrating the physical-chemical stability of PHBV films in such conditions of reuse. In the case of ethanol 50% (*vol*/*vol*), OM was not impacted during the first dishwashing cycles, with a value of about 3.5 ± 0.6 mg.dm^−2^. This OM value was lower than in ethanol 95% (*vol*/*vol*), which was in agreement with previous results demonstrating that ethanol 95% (*vol*/*vol*) was the worst case for PHBV [20]. From 50 cycles of dishwashing, OM values in ethanol 50% (*vol*/*vol*) significantly increased, becoming equal or greater than OM values in ethanol 95% (*vol*/*vol*) and higher than the OML from 100 dishwashing cycles. This could be ascribed to the release of water soluble components and/or to an increased sensitivity of PHBV films towards water upon ageing. In conclusion, considering OM values, PHBV could be considered as reusable as food contact materials up to 50 dishwasher cycles. For a higher number of dishwashing cycles, recommendations should be made regarding the type of food that can be used on contact.

The following section will focus on the impact of the incorporation of (ligno-)cellulosic fibres on the structural and physical-chemical stability of PHBV-based composites over successive cycles of dishwashing.

### 3.2. Stability of PHBV/(Ligno-)Cellulosic Fibre-Based Biocomposites

#### 3.2.1. Description of (Ligno-)Cellulosic Fibres

Three different types of (ligno-)cellulosic fibres were used in this study, i.e., Arbocel B00, Arbocel BE 600 30 PU and Filtracel EFC 950, and incorporated at 20 and 40 wt% filler content in the PHBV matrix. Those fillers were chosen for their contrasted intrinsic characteristics, i.e., cellulose content, size aspect ratio and median apparent diameter (Table 2). Arbocel B00 and Arbocel BE 600 30 PU were both quasi-pure cellulose fibres, while Filtracel EFC 950 were wood fibres recovered from a chemithermomechanical (CTMP) wood pulp, thus containing lignin, hemicelluloses and extractives. This had a direct impact on the colour of fibres, wood fibres being brown while Arbocel B00 and Arbocel BE 600 30 PU were white. This was also expected to have an impact on the final colour of films, as well as on the functional properties due to possible differences in affinity with the PHBV matrix. Moreover, the morphology of fillers, i.e., the aspect ratio L/d, filler’s dimensions and the homogeneity of filler size distribution, is a key point and can also play a role in final properties of biocomposite films. First, CTMP wood fillers seem to show heterogeneous filler size distribution with a span around 4.0 ± 0.2. Then, both Arbocel cellulosic fillers differ from each other by their aspect ratio and filler length, being higher for Arbocel B00 with L/d around 4.8 and average diameter d_50_ = 57 µm, against L/d = 2.0 and d_50_ = 31 µm for Arbocel BE 600 30 PU.

#### 3.2.2. Visual Aspect and Colour

**Figure 6:**
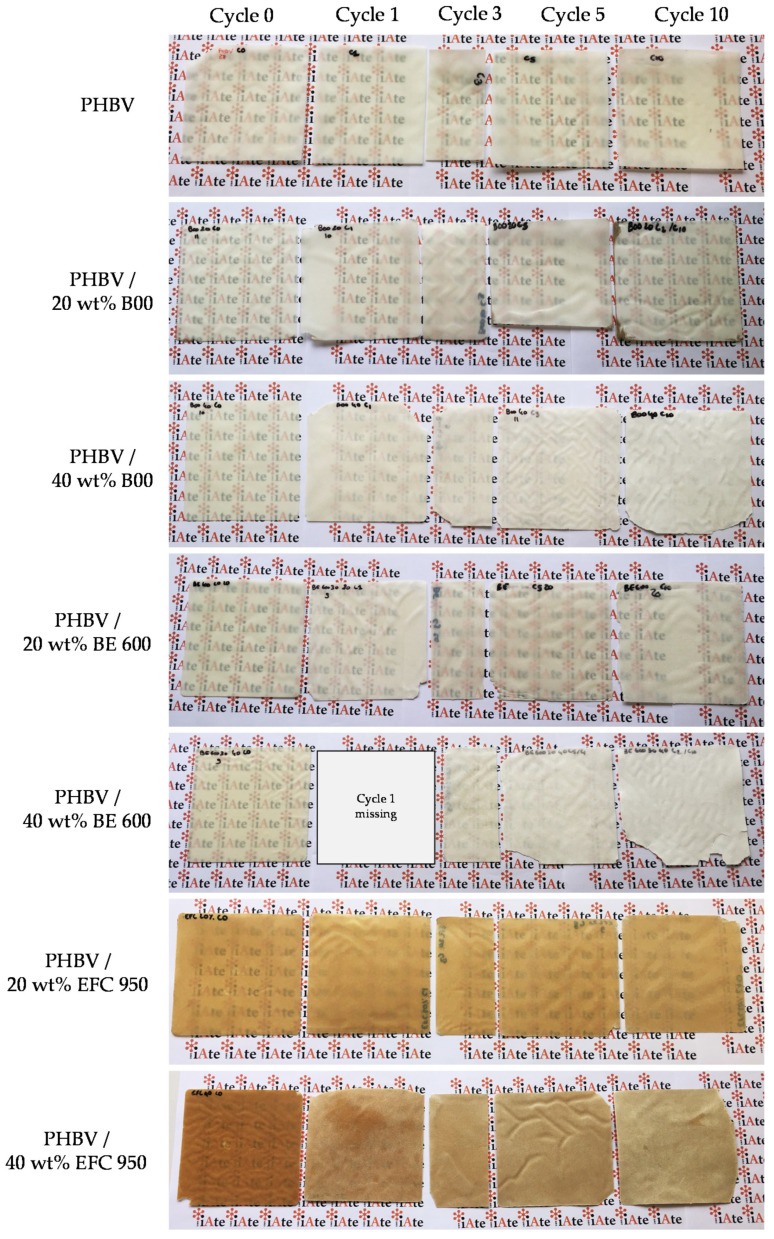
Pictures of PHBV and PHBV/(ligno-)cellulosic fillers biocomposite films at 20 wt% and 40 wt% filler content after different dishwashing cycles (0, 1, 3, 5 and 10 cycles).

Pictures of PHBV/(ligno-)cellulosic fibre-based biocomposite films are shown on Figure 6, from 0 to 10 successive dishwashing cycles. 

The incorporation of wood fillers (Filtracel EFC 950) resulted in brown coloured films due to the presence of lignin and extractives, while the colour of PHBV films was not significantly impacted by the incorporation of quasi-pure Arbocel B00 and Arbocel BE 600 30 PU cellulosic fillers. For the three types of fillers, the visual aspect of biocomposite films strongly evolved upon successive dishwashing cycles. Several observations were done. First, the surface roughness increased, with a progressive erosion of the surface. Second, missing corners and film damages were globally observed, especially after 5 and 10 dishwashing cycles, due to an increased brittleness of films. Third, increasing the filler content up to 40 wt% induced a great increase of film opacity over successive dishwashing cycles. Concerning PHBV/40 wt% EFC 950 biocomposite films, a bleaching was obvious after only one cycle and increased until 10 cycles.

Colorimetry measurements were consistent with visual observations (Table 3). Compared to the neat PHBV films, ΔE values calculated for biocomposite films were much higher, with values up to 14 after 10 cycles (far greater than the 1.4 value measured for PHBV after 125 cycles). The increase of ΔE was all the more important for increased filler content. Worth to note also that one measurement seems to be an outlier with ΔE = 15.6 for PHBV/20 wt% BE 600, probably due to wash deposits on the films. The strong evolution of the optical properties of biocomposites over successive dishwashing cycles could be ascribed to the release of low molecular weight substances such as tannins (explaining the bleaching phenomenon), changes in microstructure or crystallinity of the PHBV polymer matrix induced by the swelling of fillers and the sorption of water (explaining the increased opacity phenomenon), and/or even the release of fillers located at the surface of films. 

#### 3.2.3. Microstructure of PHBV-Based Biocomposites

**Figure 7:**
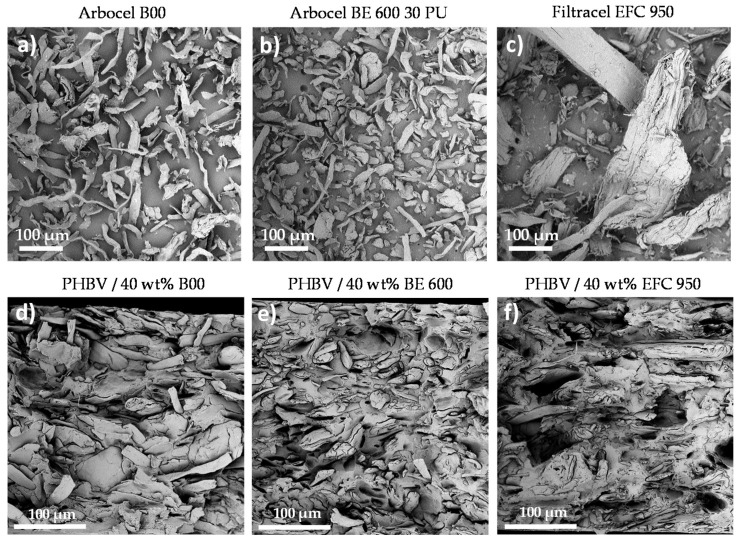
SEM pictures of (ligno-)cellulosic fillers (×500 magnification) (**a**) Arbocel B00, (**b**) Arbocel BE 600 30 PU and (**c**) Filtracel EFC 950, as well as SEM images of cryofractured cross-sections of (**d**) PHBV/40 wt% B00, (**e**) PHBV/40 wt% BE 600 and (**f**) PHBV/40 wt% EFC 950 at cycle 0 (×800 magnification).

SEM pictures were taken on (ligno-)cellulosic fillers (Figure 7a–c) and cryofractured cross-sections of PHBV-based biocomposite films filled with 40 wt% of fibres (cycle 0) (Figure 7d–f). Filtracel EFC 950 fillers (Figure 7c) were morphologically very different than Arbocel cellulosic fillers (Figure 7a,b), with a large size distribution and much bigger dimensions. Both Arbocel fillers were more similar, with more homogeneous sizes (the lower size distribution is confirmed by the lower span values), although Arbocel B00 fillers appeared to have larger average dimensions and aspect ratio, which is in coherence with filler’s characteristics detailed in Table 2.

Concerning the microstructure of PHBV-based biocomposites, (ligno-)cellulosic fillers (in light grey) seem not to provide a strong cohesive interface with the PHBV matrix. Indeed, the presence of fibre debonding, highlighted by either the presence of holes or detached fibres, and also the presence of interfacial voids around the fibres were observed (Figure 7d–f). The dispersion of cellulosic fillers (Arbocel B00 and Arbocel BE 600 30 PU) within the PHBV matrix visually seemed more homogeneous than for wood fillers (Filtracel EFC 950). In this latter case, many holes were obvious, creating preferential sites of cracks during mechanical solicitations and/or preferential pathways for the sorption of water during dishwashing (or solvent during overall migration tests). SEM images were also taken on the same PHBV-based biocomposite films having undergone 5 dishwashing cycles but no significant differences were observed (see Figure S1 in Supplementary Material).

In order to complete the analysis of the microstructure of PHBV-based biocomposite films, and especially the crystallinity of the PHBV matrix, DSC measurements were performed (Table 4). Before any dishwashing cycle, the melting temperature *T_m_* decreased with increasing content of BE 600 and EFC 950 fillers, from 175.9 ± 0.1 °C for the reference PHBV to 171.9 ± 0.4 °C for PHBV/40 wt% BE 600 and to 169.8 ± 0.9 °C for PHBV/40 wt% EFC 950 biocomposites (Table 4 and see Figure S2 in Supplementary Material). Such a decrease could be ascribed to a decrease in the molecular weight of PHBV induced by thermal degradation during the process [39,40,41]. No decrease was noticed for materials filled with Arbocel B00 fibres, showing the greater thermal stability with this type of filler. During the 125 dishwashing cycles, no change of *T_m_* was reported for PHBV films. However, *T_m_* was slightly decreased after only 10 cycles for PHBV/20 wt% EFC 950 and all PHBV-based biocomposite films at 40 wt% filler content, showing the higher sensitivity towards dishwashing of PHBV-based biocomposite films containing high (ligno-)cellulosic filler content and less pure wood fillers. 

The trends were the same considering the crystallization temperature, with *T_c_* = 127.2 ± 0.3 °C for the reference PHBV (cycle 0) decreasing down to 125.7 ± 0.1 °C for PHBV/40 wt% BE 600 and to 123.9 ± 0.4 °C for PHBV/40 wt% EFC 950 films (Table 4 and see Figure S2 in Supplementary Material). The crystallization phenomenon seems thus to be hindered by increasing the amount of low-purity (ligno-)cellulosic fillers. The crystallization phemenon was all the more delayed by increasing dishwashing cycles, and especially from 5 to 10 cycles, with crystallization peaks moved to lower temperatures. Worth to note also that in the case of materials filled with EFC 950 wood fibres, the exothermic peak was larger, highlighting the presence of bigger and more heterogeneous crystals (Figure S2f in Supplementary Material). The effect of dishwashing cycles on the crystallization phenomenon may be ascribed to the previously described water sorption capacity, increasing the swelling and the water content of fillers, which could have an anti-nucleating effect of PHBV chains.

Finally, the degree of crystallinity *χ_c_* (%) of PHBV matrix has been calculated from melting enthalpies of the first heating step (Table 4). First at cycle 0, the filler content does not seem to have a consistent effect, with degree of crystallinity values being either lower or higher than the PHBV reference value. However, differences are more significant at 40 wt% filler content with values at +25.4% or −4.0% than the PHBV reference, directly depending on the grade of (ligno-)cellulosic fillers. However, it seems difficult to understand the role of the different types of fillers with values that do not follow a clear trend. Finally, there is an impact of the dishwasher on the degree of crystallinity of PHBV with quasi-systematically an increase up to 5 dishwashing cycles and then a decrease (Table 4).

These changes in (micro-)structure and crystallinity of PHBV-based materials will impact directly their water sorption capacity, their mechanical behaviour, as well as their stability in liquid food simulants, with the creation of defects and preferential paths for the water penetration and the development and propagation of cracks.

#### 3.2.4. Weight Uptake and Swelling of PHBV-Based Biocomposites

**Figure 8:**
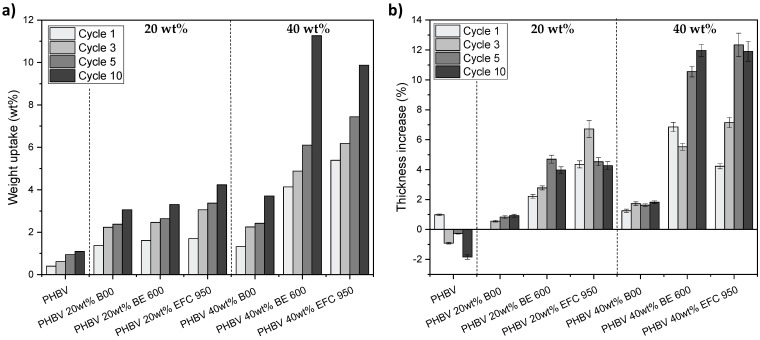
(**a**) Weight uptake and (**b**) thickness increase after 1 to 10 dishwashing cycles for PHBV and PHBV-based biocomposites filled with 20 wt% and 40 wt% of (ligno-)cellulosic fibres.

The impact of the presence of (ligno-)cellulosic fillers on the penetration level of water within the films was assessed through weight uptake and thickness measurements (Figure 8). The weight of all PHBV-based materials continuously increased over successive dishwashing cycles, which was mainly ascribed to water uptake. This weight uptake was all the more important with increasing content of (ligno-)cellulosic fillers (Figure 8a). This weight uptake was correlated to an increase of the thickness of the materials over successive dishwashing cycles (Figure 8b), except for the neat PHBV films. Indeed, the thickness increase is comprised between 0 to 6.7 ± 0.6% with 20 wt% filler content, against 1.3 ± 0.1% to 12.3 ± 0.8% with 40 wt% filler content. Such a swelling was ascribed to both the hydrophilic character of (ligno-)cellulosic fillers and the material heterogenity. In the case of neat PHBV films, the thickness increased after the first cycle and then decreased with a slight loss of 1.8 ± 0.1% of the thickness after 10 cycles. This loss was consistent with the previsouly observed surface erosion of the PHBV films.

The nature of the filler strongly impacted the sorption capacity, with materials filled with Arbocel B00 (99.5% cellulose content) sorbing much lower water compared to Arbocel BE 600 30 PU fillers (98% cellulose content) and Filtracel EFC 950 fillers (CTMP wood fillers). This effect was all the more pronounced for a filler content of 40 wt%. Similar results were obtained regarding the thickness increase. Indeed, the thickness increase for materials filled with Arbocel B00 fillers was 1.8 ± 0.1% for 40 wt% filler content after 10 dishwashing cycles, while it was around 12.0 ± 0.6% for materials filled with 40 wt% of Arbocel BE 600 30 PU and Filtracel EFC 950 fillers (Figure 8b). This result involves the importance of several parameters considering the grade of (ligno-)cellulosic fillers, such as the biochemical composition, the cristallinity rate and the morphology (length, aspect ratio, homogeneity in size). The biochemical composition and the cristallinity directly impact the intrinsic water sorption behaviour of fillers, with water sorption favoured by a low cellulose purity and a low cristallinity [42,43]. On the other hand, the morphology and the surface composition of fillers is also known to affect the filler’s dispersion state and the fibre/matrix interfacial adhesion. The formation of aggregates, agglomerates and percolation pathways, as well as a poor interacial adhesion (presence of interfacial voids around the fibres) are in favour of both water diffusion and sorption due to the presence of preferentiel pathways for the penetration of water. In the present study, Arbocel B00 and Arbocel BE 600 30 PU fillers have both a very high cellulose content but show significant differences in water sorption behaviour. This could be ascribed to differences in crystallinity. Arbocel BE 600 30 PU fillers being more amorphous than Arbocel B00 fillers, it was demonstrated to induce higher water uptake and swelling [42,43].

#### 3.2.5. Tensile Properties

**Figure 9:**
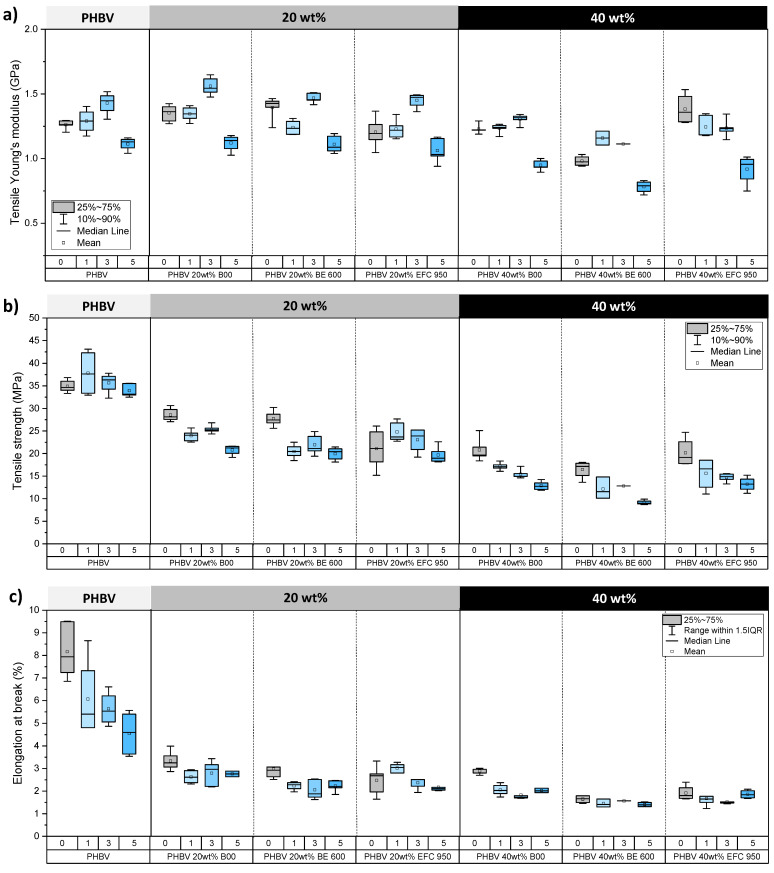
Mechanical performance of PHBV-based biocomposite films filled with 0, 20 and 40 wt% (ligno-)cellulosic fibres: (**a**) Young’s modulus, (**b**) tensile strength and (**c**) elongation at break. Films were tested after 0, 1, 3 and 5 dishwashing cycles.

Regarding PHBV/(ligno-)cellulosic fibre-based biocomposite materials, tensile tests were carried out with films having undergone up to five cycles, the films being too brittle and difficult to handle and characterise beyond five cycles. All the results are presented in Figure 9. First, worth to note that for some samples, high standard deviation values were obtained, mainly due to the possible local heterogeneities of PHBV-based composite materials and also to the fact that tensile specimens corresponsed to dumbbells punched within thermopressed films, which could cause some damages and possible early microcracks. Worth to remind also that in our case, the targeted objective was to preserve as much as possible mechanical properties, compared to the neat PHBV matrix, while decreasing the overall cost and environmental impact of the PHBV-based materials by increasing as much as possible the amount of (ligno-)cellulosic fillers.

Before going to the dishwasher (cycle 0), the Young’s modulus of the different PHBV biocomposites was maintened compared to the PHBV reference with a median value of 1.3 GPa. Indeed, a strong overlap between the different box charts was noticed, except for the PHBV/40 wt% BE 600 film showing a lower Young’s modulus of 1.0 GPa. It therefore appears that both the increase in the filler content from 20 wt% to 40 wt% and the filler’s type do not significantly influence the stiffness of films considering the overall variability. However, the number of successive dishwashing cycles had an impact on the Young’s modulus with almost a systematic increase up to 3 dishwashing cycles, and then a decrease after five cycles, except for PHBV/40 wt% EFC 950 (wood fibres) with a continuous decrease of stiffness throughout successive cycles (Figure 9a). This result seems to be more related to the PHBV polymer matrix, as the same trend was observed above for neat PHBV after successive dishwashing cycles (Figure 3b).

Regarding the tensile strength, the incorporation of (ligno-)cellulosic fibres in the PHBV matrix had a negative impact. The higher the filler content, the worst the tensile strength, mainly because of the poor fibre/matrix compatibility. Indeed, the tensile strength dropped by about 20% to 37% at a filler content of 20 wt% and by about 43% to 51% at a filler content of 40 wt%, compared to the PHBV reference with a median value of 35.0 MPa (cycle 0) (Figure 9b). Tensile strength values were also influenced by the type of fibre. However, it was difficult to conclude on the better grade considering all the formulations (Figure 9b). At a filler content of 20 wt%, cellulosic fillers (Arbocel B00 and BE 600) gave better results with tensile strength median values around 28.0 MPa, much more higher than 21.0 MPa for wood fillers. This result could be ascribed to the higher amount of cellulose in Arbocel fillers. Indeed, the biochemical composition is known to have a huge influence on tensile strength, with higher values obtained for (ligno-)cellulosic fibres containing low amounts of lignin and extractives [44,45,46]. At a filler content of 40 wt%, tensile strength values ranged from 17.0 to 19.0 MPa, with the lowest value for Arbocel BE 600 fillers. This could be ascribed to their lower aspect ratio (L/d of 2.0 for Arbocel BE 600 fillers and 4.8 for Arbocel B00). Indeed, it has already been demonstrated that the higher the fibre’s aspect ratio, the most efficient the load transfer from the matrix to the fibres, and the higher the strength of the composite [47]. It has to be precise that at 40 wt% filler content, materials are concentrated in fibres, leading to much more fibre-fibre interactions and driving to different biocomposite’s breaking mechanisms. The impact of dishwashing was clearly visible with a continuous decrease of the tensile strength throughout successive dishwashing cycles (Figure 9b), for example 20.8 MPa > 17.2 MPa > 15.5 MPa > 12.9 MPa for PHBV/40 wt% B00 and 20.2 MPa > 15.6 MPa > 14.7 MPa > 13.2 MPa for PHBV/40 wt% EFC 950, corresponding to cycle 0 > cycle 1 > cycle 3 > cycle 5. Given the quite good stability of the tensile strength of neat PHBV films during the first 5 cycles, this could rather be explained by a decrease of fibre-matrix interfacial interactions due to the swelling of water.

Finally, the elongation at break of films was strongly decreased by the addition of (ligno-)cellulosic fibres (Figure 9c), with median values between 1.7% and 3.3% against 7.9% for the reference PHBV at cycle 0. The incorporation of fillers within the PHBV polymer matrix induced defects that can initiate cracks, exacerbated by the poor PHBV/(ligno-)cellulosic fibre compatibility. Since the elongation at break values are very low, it is difficult to see the impact of the fibre content, even if it appears that the higher the fibre content, the lower the elongation at break. Moreover, it seems complicated to identify a fibre grade that stands out, even if the PHBV/B00 biocomposite films seem to give higher elongation at break values at cycle 0. Dishwashing also had a negative impact on the elongation at break from the first dishwashing cycle making the films more fragile, as seen above on film’s pictures (Figure 6). Subsequent dishwashing cycles had no significant additional impact, the elongation at break values being already very low.

To conclude, the addition of (ligno-)cellulosic fibres has a negative impact on mechanical properties of PHBV-based materials. The Young’s modulus is the least affected paramater, while properties in the rupture domain, i.e., the tensile strength and the elongation at break, are drasticlaly decreased. The dishwasher also has a bad impact on tensile properties and especially on the tensile strength of films with, for example, a drop from 46% after five dishwashing cycles for PHBV/40 wt% BE 600 biocomposite. This result is mainly due to the sorption of water in the materials favoured by the addition of very hygroscopic (ligno-)cellulosic fibres, as mentioned before (Figure 8).

#### 3.2.6. Overall Migration

**Figure 10:**
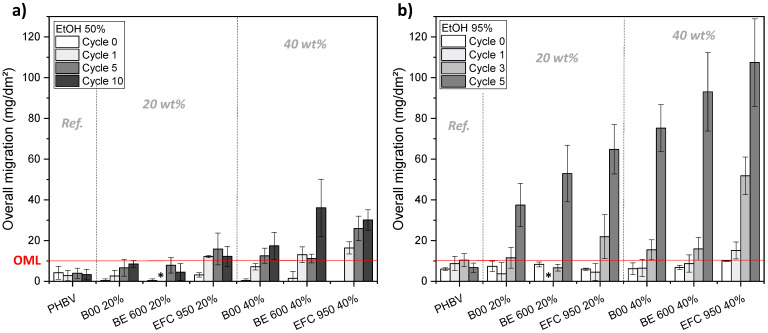
Overall migration values in (**a**) ethanol 50% (*vol*/*vol*) and (**b**) ethanol 95% (*vol*/*vol*) on PHBV and PHBV-based biocomposite films having undergone up to 10 or 5 dishwashing cycles, respectively. The red line corresponds to the overall migration limit (OML) set by the European Commission (2011) [25]. The symbol * for BE 600 20% (cycle 1) corresponds to missing values.

Overall migration tests have been performed on PHBV-based biocomposite films having undergone up to 10 dishwashing cycles (Figure 10). 

In the case of ethanol 50% (*vol*/*vol*) (Figure 10a), OM values remained lower than 10 mg.dm^−2^ for biocomposites filled with 20 wt% of cellulosic fibres, i.e., B00 and BE 600 30 PU. However, OM values were higher than the OML value for PHBV/20 wt% EFC 950 biocomposites (wood fillers) after only one cycle. At higher filler content 40 wt%, all filler’s types show OM values higher than the OML after one or five cycles, especially EFC 950 going to 26.0 ± 6.0 mg.dm^−2^ after five cycles. There is an exception with PHBV/40 wt% B00 that is still stable after one cycle. The release of components is accentuated in ethanol 95% (*vol*/*vol*) with a huge amount of liberated substances after only five dishwashing cycles with OM values going from 37.4 ± 10.6 mg.dm^−2^ to 64.8 ± 12.1 mg.dm^−2^ at 20 wt% and from 75.3 ± 11.5 mg.dm^−2^ to 107.5 ± 21.5 mg.dm^−2^ at 40 wt% filler content, as shown in Figure 10b. This strong migration in ethanol 95% (*vol*/*vol*) from zero to five cycles could be due to the degradation of PHBV matrix, as shown in Figure 5, the ethanol 95% (*vol*/*vol*) being the worst case until 50 dishwashing cycles, compared to the ethanol 50% (*vol*/*vol*). Indeed, with the PHBV matrix being damaged, the release of fillers becomes easier due to the poor (ligno-)cellulosic filler/matrix interface. The Figure 10b shows also differencies between fibre types, especially after 5 dishwashing cycles. It seems that the overall migration is highest with wood fibres, whatever the filler content, followed by Arbocel BE 600 30 PU cellulosic fillers. It therefore appears that Arbocel B00 cellulosic fillers provide lower migration into both tested food liquid simulants ethanol 50% (*vol*/*vol*) and ethanol 95% (*vol*/*vol*).

All results on overall migration tests have been summarized in Table 5 to consider possible reuse of PHBV and PHBV/(ligno-)cellulosic biocomposites as food contact materials. PHBV could be reused up to 50 or 125 dishwashing cycles, depending on the type of food. Concerning PHBV-based biocomposite materials, it appears that materials filled with quasi-pure cellulosic fibres such as Arbocel B00 show a better stability in contact food simulant liquids. However, the number of possible dishwashing cycles, from 1 to 10 depending on the (ligno-)cellulosic filler type and content, is very far from the 50 to 125 cycles reported for neat PHBV materials, making these biocomposites difficult to consider for reuse as food contact materials if a dishwashing step is carried out.

## 4. Conclusions

The aim of this work was to study the structural and physical-chemical stability of neat PHBV and PHBV biocomposite materials under dishwashing conditions, in order to consider a possible reuse of this type of materials as food contact materials. Neat PHBV films were very stable, with negligible change in appearance and colour after 125 dishwashing cycles. Regarding mechanical properties, only a slight decrease of rigidity and ductility during the first 5 cycles was noticed, then remaining stable up to 125 dishwashing cycles. Considering OM values, which is the key point to ensure food safety, they remained lower than the overall migration limit of 10 mg.dm^−2^ established by the UE Committee for the two tested food liquid simulants, i.e., ethanol 50% (*vol*/*vol*) and ethanol 95% (*vol*/*vol*), up to 50 dishwasher cycles. For a higher number of dishwashing cycles, OM values were slightly higher than the OML in the case of ethanol 95% (*vol*/*vol*), considered as the worst case for PHBV. Anyway, the present work highlights that biodegradable does not mean unresuable.

The incorporation of increasing amounts of (ligno-)cellulosic fillers negatively impacts the overall stability of PHBV-based materials. Over the 10 first successive dishwashing cycles, biocomposite films became rapidly and obviously less transparent and very brittle, with increased surface rouhghness. Bleaching was also observed for materials filled with Filtracel EFC 950 wood fibres. The loss in biocomposite performance was ascribed to the degradation of the polymer matrix through hydrolysis reactions, as well as by the swelling of hydrophilic (ligno-)cellulosic fillers, both induced by the sorption of water. It was shown that the biochemical composition, the crystallinity rate and the morphology (aspect ratio, homogeneity of the size distribution) of (ligno-)cellulosic fibres had a strong impact on the stability of PHBV-based biocomposites. High-purity cellulose fibres, with high crystallinity and aspect ratio, should be preferred to limit water sorption and the release of water-soluble components during dishwashing. In the best case, that is to say with materials filled with up to 20 wt% of cellulose fibres, up to only 10 cycles could be applied.

Worth to note that to be able to conclude on the possibility to reuse PHBV-based materials as food contact materials, several additional investigations should be carried out. First, overall migration tests should be completed with specific migration tests. It would also be necessary to include the use step and possible cross-contaminations [48] in the overall processus, i.e., to consider alternating steps of use in food contact and washing. Finally, the study if the physical-chemical stability should be coupled with the microbial stability on PHBV-based materials [35]. Once the reusability can be fully demonstrated, collection and sorting steps should be established. 

## Figures and Tables

**Table 1:** Melting temperature *T_m_* and enthalpy ∆*H_m_*, degree of crystallinity *χ_c_* and crystallisation temperature *T_c_* of PHBV films determined on the first heating and cooling steps of DSC thermograms.

		Melting Temperature *T_m_* (°C)	Composite’s Melting Enthalpy Δ*H_m_* (J.g^−1^)	PHBV’s Degree of Crystallinity *χ_c_* (%)	Crystallisation Temperature *T_c_* (°C)
**PHBV**	Cycle 0	175.9 ± 0.1	82.3 ± 1.0	56.4 ± 0.7	127.2 ± 0.3
	Cycle 5	176.6 ± 0.8	84.6 ± 0.3	57.9 ± 0.3	127.2 ± 0.1
	Cycle 10	175.2 ± 0.1	82.8 ± 0.4	56.7 ± 0.3	127.5 ± 0.1
	Cycle 50	177.6 ± 1.1	82.5 ± 0.6	56.5 ± 0.4	127.2 ± 0.0
	Cycle 70	176.6 ± 0.1	83.9 ± 0.5	57.5 ± 0.3	127.1 ± 0.2
	Cycle 125	175.9 ± 0.8	81.9 ± 0.3	56.1 ± 0.2	127.3 ± 0.1

**Table 2:** Features of the three grades of (ligno-)cellulosic particles used as fillers. Apparent diameters in volume were measured by laser granulometry.

Filler grade	Code	Type	L/d (Supplier)	d_10_ (µm)	d_50_ (µm)	d_90_ (µm)	Span	L*	a*	b*
Arbocel B00	B00	Short cellulose fibres/ 99.5% cellulose content	4.8	15.5 ± 0.1	57.1 ± 0.5	161 ± 1.7	2.55 ± 0.03	97.0	−0.33	3.86
Arbocel BE 600 30 PU	BE 600	Bleached cellulose microfibres/ 98% cellulose content	2.0	10.3 ± 0.1	31.0 ± 0.2	78.1 ± 1.1	2.19 ± 0.04	96.8	−0.51	4.62
Filtracel EFC 950	EFC 950	Purified chemithermo-mechanical (CTMP) wood pulp	2.1	17.3 ± 0.2	94.8 ± 1.9	394 ± 20	3.97 ± 0.22	76.8	4.40	22.8

**Table 3:** Difference of colour ΔE of neat PHBV and PHBV-based biocomposite films undergoing up to 10 dishwashing cycles.

		Difference of Colour ΔE		
		Cycle 1	Cycle 5	Cycle 10
**PHBV**		0.5	0.8	0.5
**20 wt%**	**PHBV/B00**	0.5	4.9	6.8
	**PHBV/BE 600**	2.6	15.6	3.1
	**PHBV/EFC 950**	0.6	1.5	3.4
**40 wt%**	**PHBV/B00**	2.7	12.4	14.5
	**PHBV/BE 600**	5.7	10.2	11.7
	**PHBV/EFC 950**	8.0	12.2	13.4

**Table 4:** Melting temperature *T_m_* and enthalpy ∆*H_m_*, degree of crystallinity *χ_c_* and crystallisation temperature *T_c_* of PHBV and PHBV-based biocomposites determined from the first heating and cooling steps of DSC thermograms, after different numbers of dishwashing cycles.

			Melting Temperature *T_m_* (°C)	Composite’s Melting Enthalpy Δ*H_m_* (J/g)	PHBV’s Degree of Crystallinity *χ_c_* (%)	Crystallisation Temperature *T_c_* (°C)
	**PHBV**	**Cycle 0**	175.9 ± 0.1	82.3 ± 1.0	56.4 ± 0.7	127.2 ± 0.3
		**Cycle 5**	176.6 ± 0.8	84.6 ± 0.3	57.9 ± 0.3	127.2 ± 0.1
		**Cycle 10**	175.2 ± 0.1	82.8 ± 0.4	56.7 ± 0.3	127.5 ± 0.1
		**Cycle 50**	177.6 ± 1.1	82.5 ± 0.6	56.5 ± 0.4	127.2 ± 0.0
		**Cycle 70**	176.6 ± 0.1	83.9 ± 0.5	57.5 ± 0.3	127.1 ± 0.2
		**Cycle 125**	175.9 ± 0.8	81.9 ± 0.3	56.1 ± 0.2	127.3 ± 0.1
**20 wt%**	**PHBV/** **B00**	**Cycle 0**	174.1 ± 0.5	65.8 ± 2.0	56.3 ± 1.7	126.6 ± 0.2
		**Cycle 5**	174.3 ± 0.5	72.9 ± 0.7	62.4 ± 0.7	126.9 ± 0.0
		**Cycle 10**	172.5 ± 0.7	67.1 ± 0.3	57.4 ± 0.3	124.8 ± 1.2
	**PHBV/** **BE 600**	**Cycle 0**	174.7 ± 0.8	68.0 ± 0.2	58.2 ± 0.2	126.4 ± 0.2
		**Cycle 5**	174.2 ± 0.6	67.3 ± 1.7	57.6 ± 1.5	126.1 ± 0.1
		**Cycle 10**	174.4 ± 0.5	62.4 ± 1.1	53.4 ± 0.9	125.9 ± 0.1
	**PHBV/** **EFC 950**	**Cycle 0**	173.7 ± 0.3	64.0 ± 7.1	54.8 ± 6.0	124.4 ± 0.4
		**Cycle 5**	176.5 ± 0.5	71.4 ± 0.7	61.1 ± 0.6	125.8 ± 0.0
		**Cycle 10**	170.5 ± 1.4	61.0 ± 6.8	52.2 ± 5.9	120.0 ± 2.8
**40 wt%**	**PHBV/** **B00**	**Cycle 0**	175.6 ± 0.4	71.7 ± 3.1	81.8 ± 3.6	126.7 ± 0.2
		**Cycle 5**	173.1 ± 0.3	60.9 ± 0.1	69.5 ± 0.1	125.1 ± 0.7
		**Cycle 10**	169.1 ± 0.4	45.4 ± 0.9	51.8 ± 1.1	121.1 ± 0.3
	**PHBV/** **BE 600**	**Cycle 0**	171.9 ± 0.4	47.0 ± 0.6	53.7 ± 0.6	125.7 ± 0.1
		**Cycle 5**	171.6 ± 0.2	53.9 ± 0.1	61.5 ± 0.1	124.4 ± 0.0
		**Cycle 10**	168.8 ± 0.3	42.8 ± 0.7	48.9 ± 0.8	120.4 ± 0.4
	**PHBV/** **EFC 950**	**Cycle 0**	169.8 ± 0.9	47.3 ± 0.7	54.0 ± 0.8	123.9 ± 0.4
		**Cycle 5**	171.6 ± 0.5	52.4 ± 0.3	59.8 ± 0.4	123.2 ± 0.5
		**Cycle 10**	168.1 ± 1.6	45.0 ± 2.6	51.4 ± 2.9	115.5 ± 2.2

**Table 5:** Admitted number of dishwashing cycles considering the overall migration limit (OML) of 10 mg.dm^−2^ set by the European Commission (2011) [25], in ethanol 50% (*vol*/*vol*) and ethanol 95% (*vol*/*vol*) on PHBV and PHBV-based biocomposite films having undergone up to 125 and 10 dishwashing cycles, respectively.

	EtOH 50% (*vol*/*vol*)		EtOH 95% (*vol*/*vol*)	
PHBV	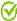 Up to 125 cycles		50 cycles	
PHBV Biocomposites	20 wt%	40 wt%	20 wt%	40 wt%
Arbocel B00	Up to 10 cycles	1 cycle	1 cycle	1 cycle
Arbocel BE 600 30 PU	Up to 10 cycles	NOT REUSABLE	3 cycles	1 cycle
Filtracel EFC 950	NOT REUSABLE	NOT REUSABLE	1 cycle	NOT REUSABLE

## Data Availability

Data supporting results can be found in the following publicly archived dataset https://doi.org/10.15454/5VJYCO (accessed on 23 December 2021).

## Supplementary Materials

The following are available online at https://www.mdpi.com/article/10.3390/membranes12020127/s1, Figure S1: SEM images of cryofractured cross-sections of PHBV/(ligno-)cellulosic fibre-based biocomposite films PHBV/40 wt% B00, PHBV/40 wt% BE 600 and PHBV/40 wt% EFC 950 before the dishwasher (cycle 0) and after 5 dishwashing cycles (×1500 magnification), Figure S2: Thermograms obtained by Differential Scanning Calorimetry of the different prepared PHBV and PHBV-based biocomposite materials at 20 wt% and 40 wt% filler content and after 0 or 10 dishwashing cycles: (a,c,e) first heating scan and (b,d,f) cooling scan.
