# Supplementary material for: Physical-Chemical and Structural Stability of Poly(3HB-co-3HV)/(ligno-)cellulosic Fibre-Based Biocomposites over Successive Dishwashing Cycles
Source: Membranes (Basel). 2022 Jan 21;12(2):127. doi: 10.3390/membranes12020127 (PMC8876846; doi:10.3390/membranes12020127)
Supplement: Supplementary file 1 [file membranes-12-00127-s001.zip › membranes-1548468-supplementary.pdf]

Supplementary Materials

Physical-Chemical and Structural Stability of Poly(3HB-co-3HV)/(ligno-)cellulosic Fibre-Based Biocomposites over Successive Dishwashing Cycles

Estelle Doineau, Fleur Rol, Nathalie Gontard and Hélène Angellier-Coussy *

JRU IATE 1208, INRAE, Montpellier SupAgro, University of Montpellier, CEDEX 02, 34 060 Montpellier, France; estelle.doineau@umontpellier.fr (E.D.); fleur.rol15@gmail.com (F.R.); nathalie.gontard@inra.fr (N.G.)

* Correspondence: helene.coussy@umontpellier.fr

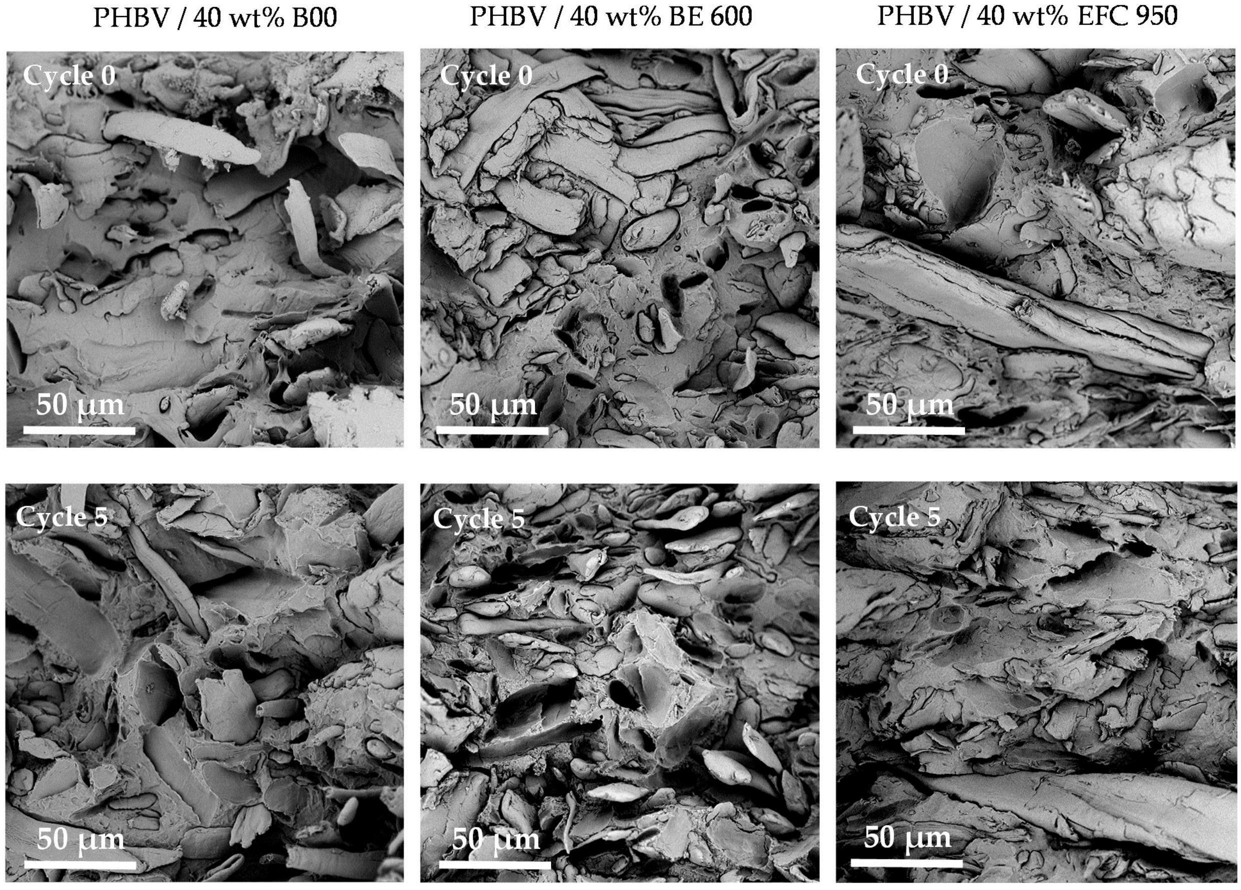

Figure S1: SEM images of cryofractured cross-sections of PHBV/(ligno-)cellulosic fibre-based biocomposite films PHBV/40 wt% B00, PHBV/40wt% BE 600 and PHBV/40 wt% EFC 950 before the dishwasher (cycle 0) and after 5 dishwashing cycles (×1500 magnification).

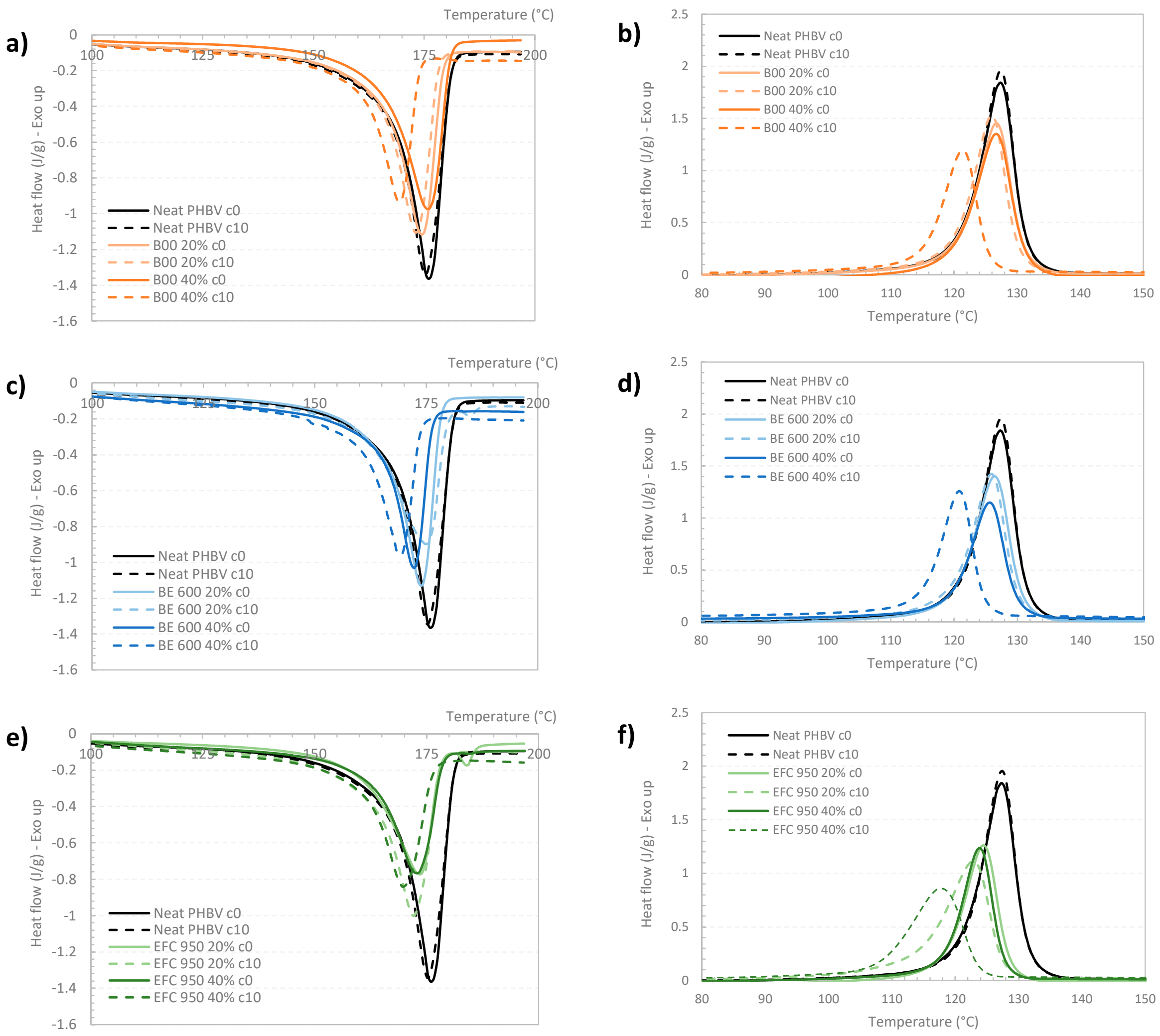

Figure S2: Thermograms obtained by Differential Scanning Calorimetry of the different prepared PHBV and PHBV-based biocomposite materials at 20 wt% and 40 wt% filler content and after 0 or 10 dishwashing cycles: (**a**,**c**,**e**) first heating scan and (**b**,**d**,**f**) cooling scan.
